# The Basic Requirement of Tight Junction Proteins in Blood-Brain Barrier Function and Their Role in Pathologies

**DOI:** 10.3390/ijms25115601

**Published:** 2024-05-21

**Authors:** Sophie Dithmer, Ingolf E. Blasig, Paul A. Fraser, Zhihai Qin, Reiner F. Haseloff

**Affiliations:** 1 Leibniz-Forschungsinstitut für Molekulare Pharmakologie, Robert-Rössle-Str. 10, 13125 Berlin, Germanyiblasig@berlincures.com (I.E.B.); 2 Kings College London, Strand, London WC2R 2LS, UK; paul.fraser@kcl.ac.uk; 3 Institute of Biophysics, Chinese Academy of Sciences, Beijing 100049, China

**Keywords:** tight junction proteins, blood-brain barrier, tight junction structure, cerebral disease, claudins, occludin

## Abstract

This review addresses the role of tight junction proteins at the blood-brain barrier (BBB). Their expression is described, and their role in physiological and pathological processes at the BBB is discussed. Based on this, new approaches are depicted for paracellular drug delivery and diagnostics in the treatment of cerebral diseases. Recent data provide convincing evidence that, in addition to its impairment in the course of diseases, the BBB could be involved in the aetiology of CNS disorders. Further progress will be expected based on new insights in tight junction protein structure and in their involvement in signalling pathways.

## 1. Introduction

Endothelial and epithelial cells separate tissues from each other and from internal and external influences by regulating the fluxes of solutes and xenobiotics; their function is indispensable to life in higher organisms. The plasma membranes of neighbouring cells are linked, so restricting lateral diffusion results in polarised cells with polarised membranes, thus generating structural and functional differences in their apical and basolateral portions [1].

Cell-cell contacts consist of tight junctions (TJs), adherens junctions, gap junctions, and (in epithelia only) desmosomes [2]. The TJ function is essential for tissue borders, such as those in the liver, kidney, or brain. Adherens junctions (AJ) are formed by cadherins [3] and stabilise intercellular contacts by connecting adjacent cytoskeleton arrays [4]. AJ are involved in the development and maintenance of cell barriers [5] and support the formation of TJs [4]. TJs and AJs form mixed contacts in endothelia, are largely indistinct in the brain [6] (whilst appearing separate in most epithelia), and are termed the apical junctional complex [7]. In gap junctions, connexins establish intercellular channels that provide intercellular communication and coordination by exchanging ions, messengers, and small metabolites [8].

**Figure 1:**
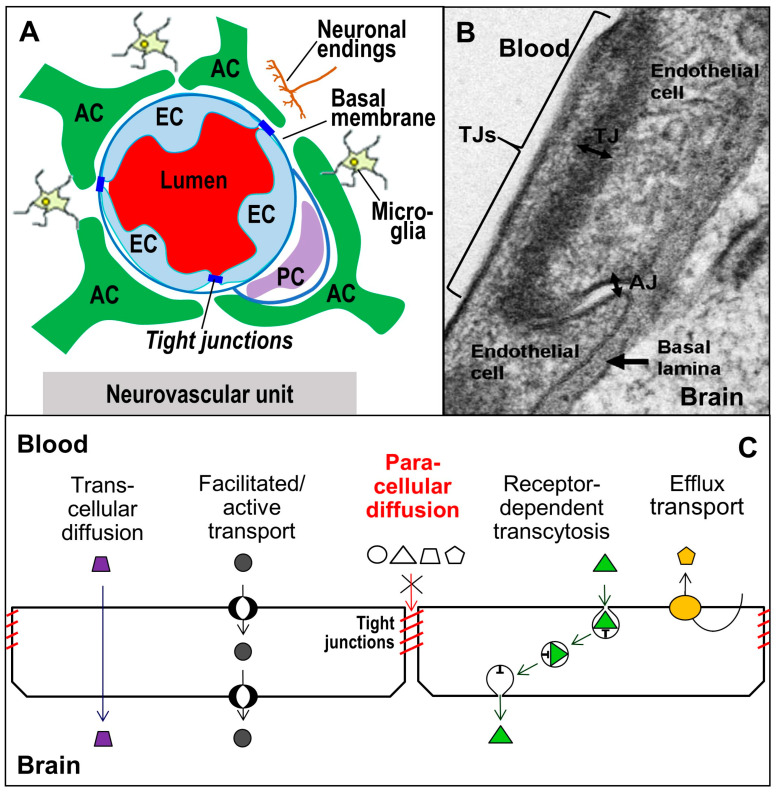
**The blood-brain barrier (BBB).** (**A**) The BBB is formed by capillary endothelial cells (EC) under the influence of other neurovascular components, such as astrocyte endfeet (AC) and pericytes (PC). (**B**) Tight junctions (TJs) seal the gap between ECs. AJ, adherens junction [9]. (**C**) Routes of passage at the BBB. TJs virtually prevent paracellular diffusion (red) and mandate transcellular transport (plasma membrane/cytosol, purple), active/facilitated transport (transporters/membrane channels, black), receptor-mediated transcytosis (green), or activation of efflux transporters (orange).

Tissue barrier function is also determined by paracellular channels, influx and efflux transport systems, transcellular pathways, metabolic regulation, and by the cellular neighbourhood. Individual barriers vary considerably in tightness and molecular selectivity depending on the expression of junctional proteins and permeability factors. The presence of TJs is the principal determinant of barrier and passage mechanisms. The exact mode of interactions of TJ proteins and their involvement in signalling pathways is far from being well understood. The processes that determine the balancing of tightness and selective permeation will be discussed to contribute to a better understanding of normal barrier function and barrier-related pathologies. This review focuses on transmembrane TJ proteins, on their structure, expression, regulation, and interactions in tissue borders. The principles are exemplified by well-studied brain barriers, in particular the blood-brain barrier (BBB, Figure 1). In this context, we also address TJ protein modulators and their potential pharmacological use.

## 2. Tight Junctions: Proteins, Functions, and Structures

**Figure 2:**
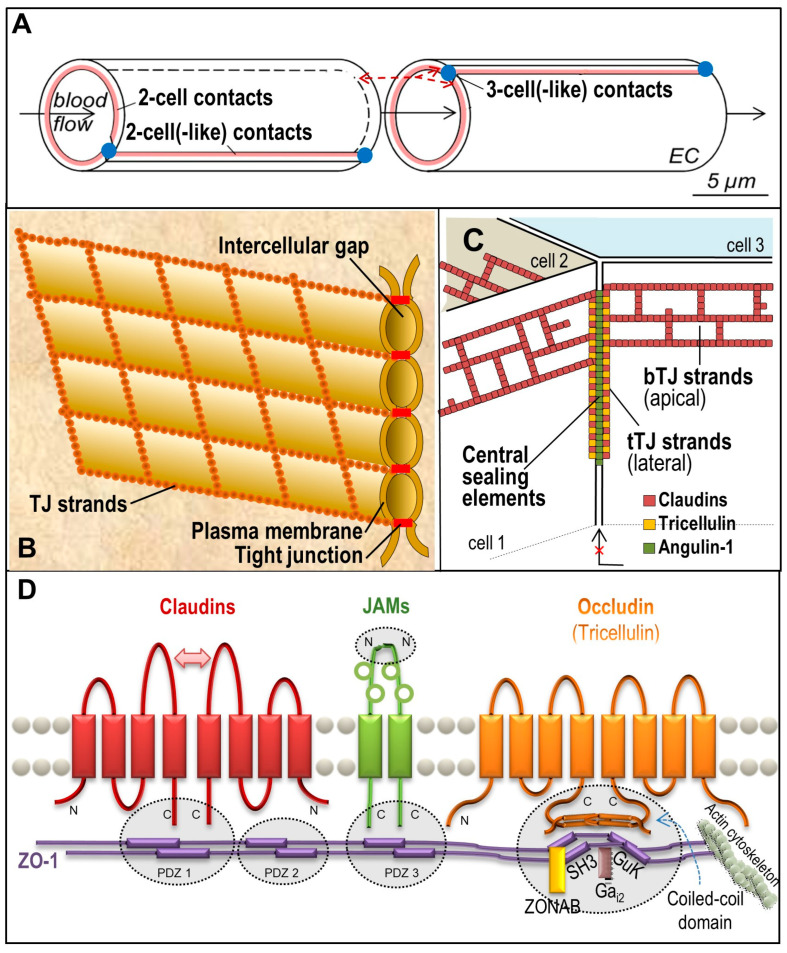
**Scheme of tight junctions (TJs).** (**A**) Longitudinal and circular TJ areas at the most luminal part of the lateral plasma membrane of brain capillary endothelial cells (EC). (**B**) Paracellular sealing by transmembrane TJ proteins forming a belt-like strand network of neighbouring plasma membranes. (**C**) Tricellular TJs as formed by non-endothelial cells (bicellular TJ, bTJ; tricellular TJ, tTJ). (**D**) Protein interactions at the TJs: claudins, occludin, JAMs, and tricellulin oligomerise along the plasma membrane and between two and/or three cell (-like) membranes. These proteins are recruited by the scaffolding protein ZO-1. Self-association occurs extracellularly (claudins), N-terminally (JAMs), C-terminally (occludin), or via PDZ2 domain (ZO-1). C-termini bind to PDZ1 (claudins), PDZ3 (JAMs), or to the SH3-Hinge-GuK unit (occludin) of ZO-1. The SH3-Hinge-GuK unit of ZO-1 also interacts with regulatory molecules, such as guanine nucleotide-binding protein G(i) subunit alpha-2 (Gα_i2_) or Y-box transcription factor ZONAB (ZO-1-associated nucleic acid binding protein).

**Figure 3:**
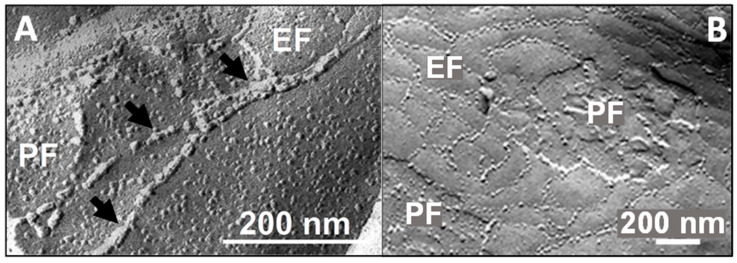
**Freeze fracture electron microscopy of tight junction (TJ) strands.** Strand networks between mouse brain capillary endothelial cells. TJ strands on exoplasmic (E-face, EF) and protoplasmic (P-face, PF) fracture of the plasma membrane indicated by arrows. (**A**) Cell culture model [49]. (**B**) Isolated brain capillaries [53].

Tight junctions (TJs) are apical cell contacts (Figure 1B) formed from protein strand networks between neighbouring plasma membranes [2] (Figure 2B). These strands appear in freeze fracture electron micrographs as closely spaced particles (diameter ~10 nm, Figure 3) which, in some tissues, provide a seal against ions, proteins, immune cells, and toxic or pharmaceutical compounds [10,11,12]. In many epithelia and endothelia, the TJ configuration allows a size- and charge-dependent paracellular diffusion for molecules or ions [13,14]. For example, in the brain, the blood-brain barrier is much denser than the blood-cerebrospinal fluid barrier [15]. Similarly, the epithelium in the renal tubule system varies considerably from leaky proximal tubules to the tight loop of Henle and collecting ducts [16]. The TJ network is arranged similarly to a belt in adjacent cell membranes (Figure 2B). The transmembrane proteins of the TJs (Figure 2D) provide the basis for differences in apical and basolateral membrane composition [17] (membrane polarity), which limits the lateral diffusion of lipids and proteins [18]. Moreover, in addition to their sealing function, TJs contribute to the regulation of cellular proliferation and differentiation [19].

There are more than 30 transmembrane proteins that are involved with TJ composition, including members the claudin family [20,21], TJ-associated MARVEL proteins (TAMPs) [22], and junctional adhesion molecules (JAMs) [23]. They are connected to intracellular structures via a number of cytosolic adaptor proteins, such as the *zonula occludens* (ZO) proteins [24], which provide a link to the cytoskeleton (Figure 2D). JAMs and ZO proteins alone do not form TJs. The transmembrane TJ proteins are subject of continuous turnover, in most cases via clathrin-mediated endocytosis [25,26], caveolin [27], or macropinocytosis [28] with subsequent lysosomal decomposition or recycling to the membrane [27,29,30].

Claudins, a multigene family of 27 members with a molecular mass of 20–29 kDa [31] (first described in 1998 [20]), form the key component of the TJs and are essential for paracellular sealing of tissue barriers [13,32] (Figure 2). Based on sequence comparison, claudins can be divided into (homologous) classic and (less homologous) non-classic claudins. A functional distinction can be made between sealers (e.g., claudin-5) and sealers that simultaneously form paracellular channels (e.g., claudin-15). The function of certain claudins is unclear (e.g., claudin-25–27) or described controversially (e.g., claudin-4, Table 1).

**Figure 4:**
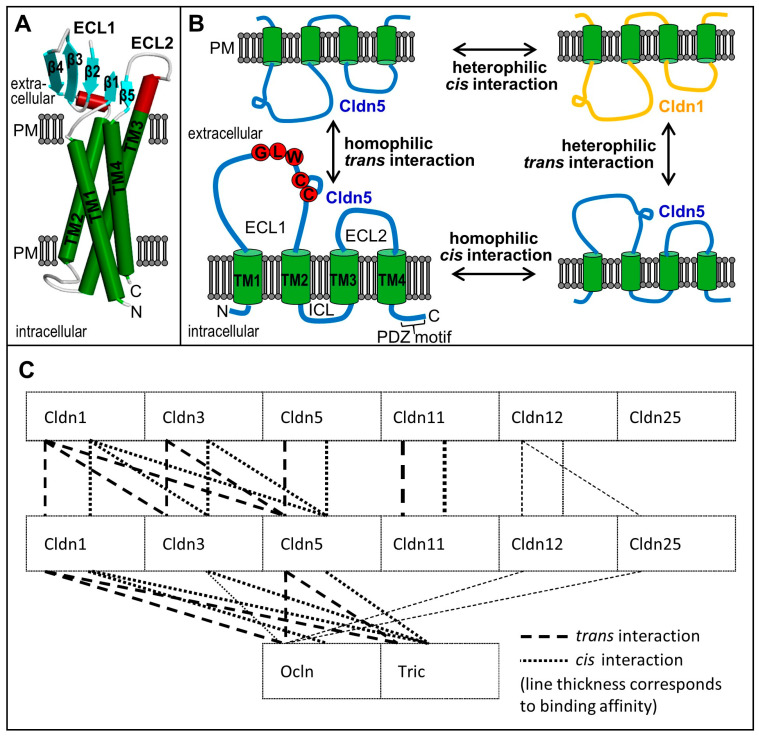
**Structural and interaction models of claudins.** (**A**) General model based on crystal structure of claudin (Cldn)-15 ([33], C-terminus truncated by 33 amino acids). ECL1 and -2 form an extracellular domain consisting of a β-sheet (five antiparallel β-strands, blue) and two α-helices (red) and unstructured areas (grey). TM, transmembrane domain; ECL/ICL, extra-/intracellular loop; PM, plasma membrane. (**B**) *Trans* interactions between claudins of neighbouring cell membranes and *cis* interactions along a plasma membrane. Red, conserved residues including two cysteines form an intramolecular disulfide bridge. PDZ, binding motif for PDZ1 domain in *zonula occludens* proteins. (**C**) Scheme of different interactions of blood-brain barrier-expressed claudins, occludin, and tricellulin [50,55].

Crystal structural data (obtained for claudin-15 [33], -19 [34], -4 [35], and -9 in complex with a toxin [36]) show a bundle of the four transmembrane helices and a joint extracellular domain consisting of a β sheet (five β-strands) and usually two short helices (Figure 4A, Supplementary Figure S1). Two cysteines in the first extracellular loop (ECL1) and the disulfide bridge included therein are part of a consensus sequence (G-L-W-x-x-C-[7–9 polar/charged amino acids]-C) [13,37,38]. In their C-terminal part, many claudins (e.g., Claudin-1–8) contain a conserved binding motif (Y/L/F/P)-x-K/R/L/V-K/R/T/S-x-Y-VCOOH [39], enabling association with the PDZ1 domain of ZO proteins [40,41] (Figure 2D). There are several sites of post-translational modification, e.g., at the conserved Tyr of the PDZ-binding motif (Table 2), that regulate claudin functions, including oligomerisation, transport processes, interactions, subcellular localisation, and homoeostasis [42]. Frequently, tyrosine is phosphorylated at the conserved PDZ binding motif [43]. Cys-palmitoylation at the intercellular loop or at the cytosolic C-terminal portion is essential for building the TJs [44].

The ultimate function of a TJ barrier depends on a number of parameters, such as cell type, tissue, organ, or even species, in addition to the role of any individual claudin and the presence and stoichiometry of other family members. For example, rat cholangiocytes or human-colon-derived Caco-2 cells express a large but considerably different subset of claudins [45]. Claudin-11 is expressed in oligodendrocytes to form the electrical seal of the myelin sheet around nerve fibres and forms the blood-testis barrier in Sertoli cells [46,47]. Claudin-13 has been found in mouse but not, so far, in human tissues (Supplementary Figure S1). The claudin profile is dynamically regulated during development and by environmental conditions (reviewed by refs. [48,49] and others). It should be noted that expression data collected in vitro often differ from those collected in vivo [50], similar to mRNA and protein expression levels. Interestingly, claudins (1, 7) have also been found at the basolateral membrane (but with unknown local function) [51,52].

TJ structures are mainly formed via oligomerisation of the extracellular claudin domains of adjacent cell membranes [54]. These *trans* interactions create—in conjunction with *cis* associations along the cell membrane (Figure 4B)—TJ strands (Figure 3), which are responsible for paracellular sealing and channel function (Table 1). Certain combinations of heterophilic interactions in *cis* and *trans* are preferred, while others do not occur. Claudin-1, -3, and -5 can interact in *cis* and *trans* with each other, claudin-2 and -11 only interact with themselves (Figure 4C). Thus, levels of the different claudin subtypes localised at the TJs determine the permeability and specificity of the paracellular pathway [49].

**Table 1:** Claudin (Cldn) functions in tight junctions.

Class	Paracellular Sealing	Function Paracellular Sealing/Channel-Forming	Other	Not Clear
classic *	Cldn1 [56]	Cldn2 [57]/(Na^+^, K^+^) [58]		
	Cldn3 [59]			
	Cldn4 [60]	Cldn4 [61]/Na^+^ [62]		
	Cldn5 [63]	Cldn7 [64]/Na^+^ [65]		
	Cldn6 [66]	Cldn10 sealing/-10a an^-^, -10b cat^+^ [67]	Cldn6 [68]	
	Cldn8 [69]	Cldn15 sealing/Na^+^, K^+^ [62]		
	Cldn9 [66]	Cldn17 sealing/an^-^ [70]	Cldn9 [68]	
	Cldn14 [71]			
	Cldn19 [72]			
non-classic	Cldn11 [73]	Cldn16 sealing/cat^++^ [72]	Cldn13 [68]	Cldn12, -13, -20 [50]
	Cldn18 [74]	Cldn21 sealing/Na^+^, K^+^, solutes ≤ 0.56 nm [75]		Cldn22, -23, -24 [50]
	Cldn25 indirectly [76] via structure of TJ [50]			Cldn25, -26, -27 [50]

*, high sequence homology [13]; an, anion; cat, cation.

**Table 2:** Characteristics of selected claudins (Cldns) of the blood-brain barrier.

Expression	Function	Structure/Interactions	Regulation/Signalling
**Claudin-1** (*Senescence-associated epithelial membrane protein*)			
- gene *CLDN1*, chromosome 3 (human), -16 (mouse) - protein: human [77,78], mouse [78,79] - cell membranal at TJs [80] and cytoso- lic [50] localisation [53] - KO mouse: postnatal dehydration, lethal [81]	- causes tightness (TER) [56,82], sealing [56,82,83] - receptor for hepatitis C- virus [84]	- 211 aa; M.W., 22.7 kDa; pI, 8.41; N-/C-terminal tail, 7/27 aa; ECL1/ECL2, 53/27 aa (human) - homophilic *cis*/*trans* interactions [85,86], dissociation constant ECL1 to Cldn1 47 ± 0.6 nM [86] - heterophilic *cis* Cldn3, -5 [87], Ocln, Tric, MD3 [55], PDZ1 of ZO-1 [88]; *trans* Cldn3,-5 [87], Ocln, Tric, [55] - continuous P-face TJ-strands [55] - low membrane mobility [55]	- GPR30 via ERK and/or Akt-domain [89] - dehydroepiandrosteron/Gnα11 [90] - hypoxia inducible factor-complex [91] - cAMP/PKA, down-regulation and cytosolic localisation [92] - down-regulated by hypoxia, focal cerebral ischemia [50], glioblastoma [93] - down-regulated by TGFβ [93], Cu [94], miR212/132 [95] - differentiated regulation upon virus infection [96,97,98,99,100]
**Claudin-3** (*Clostridium perfringens enterotoxin receptor* 2)			
- gene *CLDN3*, chromosome 7 (human), 5 (mouse) - protein: human [77,78], mouse [78,79], rat [101] - KO mouse: amount of Cldn5 and Ocln, paracellular permeability reduced [53]; no changes found by other authors [102]	- enhances BBB integrity in vivo [78], increases complex-ity of TJ-strand network [53] - controls paracellular tightness [59,103,104] (particularly small molecules/ions) - limits endocytosis; pro-motes infarction/oedema [53] - supports embryogenesis/ postnatal development, stabilises BBB/TJ [105]	- 220 aa; M.W., 23.3 kDa; pI, 8.37; N/C-terminal tail, 8/40 aa; ECL1/ECL2, 51/23 aa (human) - homophilic interaction *cis*/*trans* [87] - heterophilic *cis* Cldn1, -5 [87], Tric, MD3 [55], associates ZO-1-PDZ1 [40]; *trans* Cldn1, -5 [87], Tric, MD3 [55] - continuous P-face strands [87,106] - high membrane mobility >Cldn5 [87] - strengthens TJ strand network/-branching [53]	- Wnt/β-catenin controlled barrier development [105] - expression modulated by Na/K-ATPase [107] - down-regulated by hypoxia/middle cerebral artery occlusion [50] - down (haemorrhage) (PI3K, sphingosine 1-phosphate receptor 1) [104] - loss in EAE, glioblastoma [78] - down-regulated at low Cu [94]
**Claudin-5** (*Transmembrane protein deleted in velocardiofacial syndrome*)			
- gene *CLDN5*, chromosome 22 (human), 16 (mouse) - very high expression [50,78,108], embryonically starting with cerebral angiogenesis [109] - KO mouse: abnormal TJs, brain capillaries permeable for molecules < 800 Da, lethal 10 h postnatally [63] - KD: BBB breakdown in tissue culture, human BEC [110] - t_1/2_ 70 min [111] - protein amount: Cldn5 > −25, Ocln, Cldn1 > −11, −12 (isolated brain capillaries, TX-100 extract) [50]	- causes paracellular tightness for molecules < 800 Da [63] - induces/maintains TJ tightness [21,112] mediated via ECL1 [113] and ECL2 [54]	- 218 aa; M.W., 23.1 Da; pI, 8.25; N-/C-terminal tail, 7/38 aa; ECL1/ECL2, 53/16 aa (human) - homophilic *cis*/*trans* interaction [54,87] - heterophilic *cis* Cldn1, -3 [87], Tric, MD3; *trans* Cldn1, -3, Ocln, Tric [55] - discontinuous E-face-associated TJ-strands (in TJ-free cells) [21,54] - low membrane mobility [87] - mixed E-/P-face strands by Cldn3 [87] - associates ZO-1-PDZ1 [40] - no effect on ZO-1 clustering [114] - C54S, C64S (mouse ECL1, aa exchange) weaken barrier [113] - ECL1-G60R, human channelopathy: Cl^-^/small molecule flux [115] - transferred from BEC to leukocytes in EAE, possibly supporting transmigration into CNS [116]	- Thr(207)-phosphorylation opens porcine BBB, protein kinase A [117] - TGF-β/activin signalling increases Cldn5 [118] - VE-cadherin via Akt-activation: phosphorylation of FoxO1 induces Cldn5 [119] - adrenomedullin: enhanced expression and TER, decreases permeability [112] - increase (mRNA, protein, promoter): gluco-corticoids TER up [120,121], estrogen [122] - ROCK via EphA2: down-regulation [123] - ROCK up in dementia: Cldn5 down [124] - C/EBP-α (stimulated by JAM-A) up-regulation, reduced permeability [125] - serum Cldn5 up: autistic children [126], severe stroke [127] -down-regulated by EphA4/Tie2/Akap12 signalling mediating microvascular dysfunction and trauma [128] - down-regulated at low Cu [94] - oxidative stress inhibitor improves Cldn5, ZO-1, TER via Nrf2/HO-1 [129]
**Claudin-11** (*Oligodendrocyte-specific protein*)			
- gene *CLDN11*, chromosome 3 (human), 3 (mouse) - mRNA/protein: very high expression in BEC (human, mouse, rat) in vivo equal to Cldn5, in vitro strongly down-regulated [50,130] - less expressed in human brain oligodendrocytes [50] - KO mouse: mild neurological deficits [131], deafness (low endocochlear potential) [47] - KD: enhanced dextran permeability through BEC layers [130]	- contributes to tightness of BEC layers [50,130] and BBB [132]	- 207 aa; M.W., 22.0 Da; pI, 8.22; N-/C-terminal tail, 1/29 aa; ECL1/ECL2, 50/14 aa (human) - very strong homophilic *cis*/*trans* interaction (Cldn11 >> other Cldns, Ocln, Tric [55,133]) - no heterophilic binding [55]; Cldn5 colocalisation in junctions [50,130] - continuous P-face oriented TJ-strands, modulated by Ocln [50] - very low membrane mobility <other Cldns [50], Ocln, Tric, MD3 [55]	- reduced in multiple sclerosis [130] - decreased in EAE by activated annexin A2 signalling (brain capillaries) [134] - decrease in BEC by podocalyxin KD [135] - increased in blood of human autism spectrum disorder [126] - ischemia reduces Cldn11; KO of leucine-rich alpha-2 glycoprotein 1 improves Cldn11 and BBB in ischemia [132]
**Claudin-12**			
- gene *CLDN12*, chromosome 7 (human), 5 (mouse) - in BEC [63,94,136]; mRNA in vivo > in vitro [50] - expressed at TJs [50,63] - lack of Cldn12: intact BBB; neurological/behavioral changes [137] - knock-in mouse: mRNA in BEC, pericytes, oligodendrocytes, smooth muscle cells, astrocytes [137]	- not crucial in establishing or maintaining BBB TJ integrity [137]	- 244 aa; M.W., 27,1 kDa; pI, 8.80; N-/C-terminal tail, 10/49 aa; ECL1/ECL2, 56/18 aa (human) - homophilic: no *cis*- [87], but weak *trans* interaction [55] - heterophilic: weak *trans* interactions with Cldn22, -24, -25, Ocln [50] - no C-terminal PDZ-binding motif [87] - no strand formation [87], very high paracellu-lar flux in TJ-free cells [138]	- ouabain-activated Na/K-ATPase reduces expression [107] - high-energy diet decreases mRNA-, increases hippocampal permeability [139] - hyperammonia reduces mRNA in vitro [140] - down-regulated in hypoxia/ischemia [50,53] and in diet-induced diabetes (in latter case attenuated by carbonic anhydrase inhibitor [141]) - regulated by Cu exposure [94]
**Claudin-25** (*Claudin domain-containing protein* 1)			
- gene *CLDND1,* chromosom 3 (human), 16 (mouse), - very high mRNA expression in vivo in BEC [50] - in human BEC localised at TJs [76] - KD: reduces mRNA/protein without cytotoxicity, paracellular permeability raises for small molecules [76]; P-face strands less structured, reduced mesh number, i.e., less particles, larger meshes] [50].	- contribution to cell adhe-sion and tightness for small molecules [76]	- 229 aa; M.W., 25.4 Da; pI, 5.37; N-/C-terminal tail, 10/44 aa; ECL1/ECL2, 50/19 aa (human) - no homophilic *trans* interaction in BEC [50] - weak heterophilic *trans* interaction (Cldn12, -22, -24, Ocln) [50] - no TJ strand formation, but strands supported indirectly (via Ocln) [50]	- xenobiotics-activated arylhydrocarbon-receptor [142], retinoic acid receptor-related orphan receptor α [143], and myeloid zinc finger 1 [144] increase mRNA expression - transcription inhibition by miR-124 [145] - cerebellar haemorrhage decreases expression in mouse BEC by [76]

aa, amino acids; Akt, protein kinase B; BEC, brain endothelial cells; BBB, blood-brain barrier; Cldn, claudin; C/EBP, CCAAT/enhancer-binding protein; *cis* interaction, cf. Figure 4B; EAE, experimental autoimmune encephalitis; E-face, exoplasmic-face; EphA2/4, ephrin type-A receptor 2/4; ERK, extracellular-signal regulated kinase; ECL, extracellular loop; FoxO1, forkhead box O1; G, G-protein; GPR, G-protein-coupled receptor; JAM, junctional adhesion molecule; KO/KD, knockout/-down; MD3, MarvelD3; M.W., molecular weight; Nrf2/HO-1, erythroid 2 like 2 nuclear translocation/haem oxygenase 1 signalling; Ocln, occludin; PI3K, phosphoinositide 3 kinase; P-face, protoplasmic-face; pI, (calculated) isoelectric point; PK, protein kinase: ROCK; Rho-associated protein kinase; t_1/2_, half-life; TER, transendothelial electrical resistance; TGF, transforming growth factor; TJ, tight junction; *trans* interaction, cf. Figure 4B; Tric, tricellulin; ZO-1, *zonula occludens* protein 1.

## 3. Tight Junctions and Their Proteins at the Blood-Brain Barrier

The BBB ensures the brain function by maintaining a constant cerebral milieu and is thus a decisive factor for the homoeostasis of the CNS. It provides a highly efficient exchange of nutrients and metabolites and prevents the permeation of xenobiotics, peripheral metabolites, pathogens, and blood cells [146]. The BBB is formed by capillary endothelial cells (Figure 1B and Figure 2A) influenced by the basal membrane, pericytes, (located within the basal membrane), neurons, microglia, and especially astrocytes. The whole ensemble is referred to as the neurovascular unit (Figure 1A). Pericytes surround a third of the endothelium [147] and support the formation and maintenance of the BBB. They contribute to angiogenesis and various brain functions. Microglia provide the immune defence of the brain by being activated under pathological conditions [148,149]. The endfeet of astrocytes almost completely envelop the capillaries [147], which is highly important for a functional BBB [150]. The capillaries exhibit an inner diameter of 3–5 µm. In humans, the capillary system has a total length of ~650 km and a surface of 10–20 m^2^ [151,152,153]. The cleft between the endothelial cells is closed by an intercellular network of TJ strands, preventing direct diffusion of solutes including Na^+^, K^+^, and water [154,155,156] (Figure 2B and Figure 3). Water transport is probably mainly diffusive since the measure of hydraulic conductance of 2 × 10^−9^ cm (cmH_2_O s)^−1^ is comparable to that of endothelial cell membranes [157].

The very high TJ density of the BBB results in a considerable transendothelial electrical resistance (~5 kΩcm^2^) as it lacks paracellular channels, as seen for other barriers [158] such as the blood-cerebrospinal fluid barrier (where claudin-2 creates ion channels [15]). Flux rates through the BBB are very low for hydrophilic molecules for which transporters are lacking [159]. The barrier function is supported by low pinocytosis, little vesicular transport, and no fenestration in the cells [146,160]. The supply of the brain with substrates and regulatory molecules is enabled by selective transport systems, which are essential because the paracellular route is blocked by TJs. The TJs are thus the key element of the BBB [161,162,163]. Additionally, a metabolic barrier is formed by the high activity of endothelial enzymes, such as γ-glutamyl transferase, alkaline phosphatase, glucose-6-phosphatase, catechol-O-methyl transferase, monoaminoxidase, or cytochrome P450 [164]. They collectively prevent their substrates passing into the CNS owing to their being metabolised during entry into or within the cell.

In general, TJs largely prevent passive paracellular diffusion of polar compounds, thus mandating diffusion across the plasma membrane and cytosol (O_2_, CO_2_, lipophilic molecules with molecular masses <500 Da, e.g., ethanol [165]). Other molecules require transporters or paracellular channels. For endothelial cells of the BBB, this includes cerebral Na^+^-uptake (indirectly that of water), extracellular HCO_3_^−^ (pH), and the maintenance of interstitial K^+^-concentration (Na^+^, K^+^-ATPase) in the brain [166]. Thus, the TJs allow for a number of electrochemical gradients to be established, and these provide the driving force for the transport of substances that maintain homoeostasis of the CNS. Special transporters also exist for hydrophilic substrates such as glucose, amino acids, lactate [167,168,169], neuropeptides [170,171], and biotransformed products [172]. Receptor-mediated transcytosis via the use of specific carrier proteins facilitates intake of macromolecules as insulin [173], LDL [174], transferrin [175], leptin [176], and others [177]. Additionally, TJs render efflux transporters barrier-effective by excluding small (400–600 Da) lipophilic/non-charged compounds from the brain, amounting to more than 300 metabolites, toxins, and drugs [178,179,180,181] (Figure 1C).

### 3.1. Claudins

**Figure 5:**
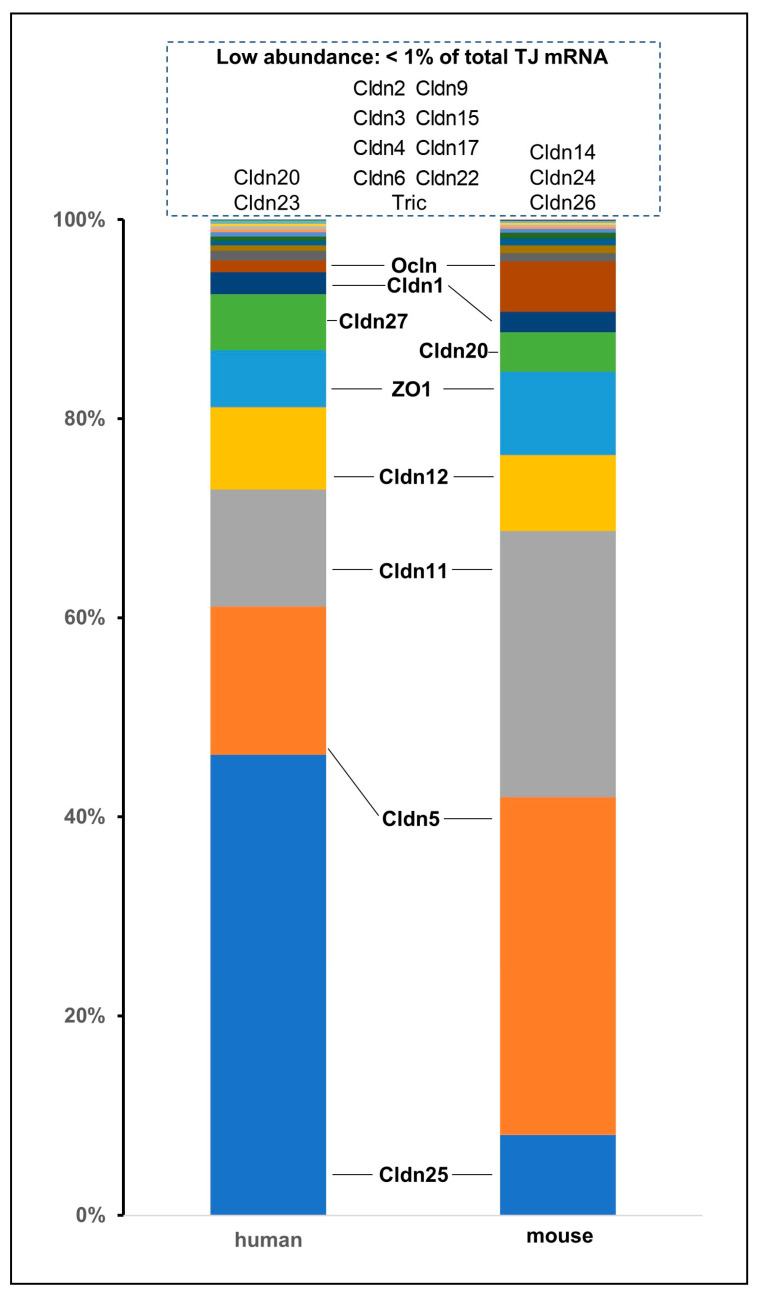
**mRNA expression of tight junction (TJ) proteins in human and mouse brain endothelium.** Proportions of individual proteins of the total expression of TJ protein mRNA. High abundance, >1%; low abundance, <1% of total mRNA ([50], modified). Cldn, claudin; Ocln, occludin; Tric, tricellulin.

Claudins 1, 5, 11, 12, 25, 27 (only human), and 20 (only mouse) are abundantly expressed in capillary endothelial cells of cerebral cryosections (mRNA). Claudins 2–4, 6, 9, 15, 17, 22, and 20 and 23 (both only human) and 14, 24, and 26 (only mouse) are less abundant [50] (Figure 5). The mRNA values roughly reflect the protein values [50,63]. Consequently, the highly abundant claudins 1, 5, 11, 12, and 25 are discussed below, while the less-abundant claudin-3 is also discussed since a number of reports suggest that it plays a role in BBB pathology [50,53,78,104,105,182] (Table 2).

Claudin-5, a tightening claudin and highly expressed [183,184], is most important for very dense TJs [158] in vertebrate BBBs [63,185]. It tightens the barrier (two-cell- or two cell(-like) contacts, Figure 2A, left) for molecules < 800 Da [63], which includes the majority of physiologically active substances. The paracellular cleft is closed via tight *cis* and *trans* interactions of the extracellular domains [115,186]. A hereditary mutation in loop 1 results in a severe channelopathy with the increased permeation of ions and small molecules [115]. These data support the great relevance of claudin-5 for the pharmacology and pathology of the BBB. Its knockdown up-regulates claudin-1 [50], pointing to a compensatory potential between sealing claudins. ZO-1 [40] and occludin [55] interact with claudin-5 and facilitate the formation of claudin-5 strands [41,55]. On the other hand, the clustering of ZO-1 is independent of Claudin-5 [114]. As visualised by freeze fracture electron microscopy, claudin-5 alone assembles in exoplasmic TJ strands [54,187]. Freeze fracture EM of mouse cerebral endothelium shows that these strands also appear on the protoplasmic surface [188] (Figure 3), which indicates that additional strand-building proteins are sufficiently expressed. In primary cultures of human brain endothelial cells, claudin-5 has a half-life of 13.8 h [189]. The trafficking is caveolin-dependent [26,27], and it is either recycled [27,30] or ubiquitinylated and degraded in the proteasome [42,111], indicating high protein dynamics.

Claudin-11, another sealing [50,130,190] protein, forms strands associated with the protoplasmic face [50], interacts only in a homophilic manner [55,133], and is almost exclusively detectable in vivo [50,133]. The protein level is comparable to that of claudin-5 [130]. Claudin-11 has a much higher homophilic affinity than claudin-5 [55], resulting in a considerably reduced membrane mobility. It can be found in distinct TJ segments being free of other claudins [50]. Molecular modelling indicates a relatively small extracellular binding domain (Supplementary Figure S1). This, combined with a strong capacity for oligomerisation, low junctional agility, and no interrelations with other claudins, suggests a very tight intercellular seal. Occludin indirectly modulates claudin-11 strand morphology [50]. Knockdown in brain endothelial cells decreases paracellular tightness [50,130]. Mild neurological deficits have been described in claudin-11 (also known as oligodendrocyte-specific protein) knockout mice, since its function in the myelin sheath is partly substituted by another structurally similar membrane protein [1,131]. Claudin-5 and -11 seem to act partly synergistically, and seem to compensate for each other; claudin-5 deficiency does not lead to a complete loss of sealing [63]. Their exoplasmic and protoplasmic strand orientations also complement each other. 

Claudin-12 is well expressed in endothelial cell contacts both in brain sections [63] and in purified brain capillary endothelial cells [136]. Claudin-12 does not, however, form TJ strands [87], probably due to its lack of homophilic *cis* interaction and a lack of the C-terminal PDZ-binding motif that prevents association with the PDZ1 domain of ZO-1 [40], which usually supports TJ formation [191]. Additionally, this claudin shows weak homo- and heterophilic [55] *trans* oligomerisation with claudin-25 and occludin. These interactions could help to maintain the support of claudin-25 for TJ strand morphology [50] or to preserve the TJ regulation by occludin [192], and it appears that claudin-12 plays a role in the maintenance of the BBB as it has been shown to be down-regulated under pathological or toxicological conditions [50,94,107,139,140,141].

Similarly, claudin-25 is highly expressed and its contribution to barrier function is unclear; it does not show homophilic *trans* interaction, which limits its potential for direct barrier sealing and TJ formation. Claudin-25 does localise at cerebral endothelial cell contacts and indirectly supports a functional TJ morphology. This is probably due to *trans* interactions with occludin [50], a main regulator of BBB TJs [192] under normal [156] and pathological [193] conditions. Its importance in TJs is demonstrated by claudin-25 knockdown, leading to the hyperpermeability of small molecules and weakening the TJ strand network in an in vitro BBB model [50]. Since claudin-25 interacts with occludin but not with strand-forming claudins, it probably indirectly contributes to TJ function. N-Glycosylation at the extracellular loop 1 of claudin-25 [194] promotes its localisation at the plasma membrane and can initiate signal transduction processes [195]. Hence, claudin-25 can be considered as a TJ modulator at the BBB.

Claudin-1 and -3 form strands associated with the protoplasmic face and are frequently involved in sealing of epithelial barriers. Their function at the BBB is not clear, their interaction potential with claudin-5, occludin, and tricellulin renders both as candidates for tightening the BBB. Claudin-1 is also considered in a developmental context [196]. Its immune reactivity is often observed as more cytosolic than junctional [50] and its deficiency does not show a cerebral phenotype [197], but overexpression has a tightening effect at the BBB [83]. The low mobility [55] correlates with strong homophilic affinity [86]. Application of claudin-1-derived peptides that block its interactions leads to higher permeability of cerebral endothelial barriers [198]. The high affinity to various interaction partners [87], including occludin [55] and its redox sensitivity [86,91,94], point to a modulatory role in corresponding pathologies.

The expression of claudin-3 at the adult BBB is rather low [50,94], and its significance for the barrier is questioned [102]. Claudin-3 knockout experiments, however, result in reduced claudin-5 expression, lower junctional occludin localisation, and increase branching of TJ strands. The strand network is weakened, and barrier permeability increases, which diminishes the infarct area and oedema formation in a stroke model [53]. This supports the idea that to attenuate ischemia-related damage (e.g., oedema) via TJ modulation using claudin inhibitors could open the BBB reversibly [199]. Changes of claudin-3 at the BBB are found in experimental ischemia/reperfusion [50,53], haemorrhage [104], and chronic inflammatory pain [182] in a multiple sclerosis model (experimental autoimmune encephalitis [130]) or in human glioblastoma multiforme [78]. Moreover, claudin-3 is involved in the development and maintenance of the BBB [105]. Thus, a role in barrier regulation is assumed, which would be supported by its high membrane mobility [87] and its interaction with other claudins and occludin [55].

In summary, claudin-5 is considered the major component of the TJs and, for small molecule tightness, the essential sealer of the BBB; claudin-11 also seals the barrier and partially compensates for claudin-5. Claudin-3 might have a limited contribution to TJ function and could be involved in pathological processes. Claudin-25 does not contribute to the structure and function of TJs directly but could modulate it indirectly. The function of claudin-12 remains unclear.

### 3.2. Tight Junction-Associated MARVEL Proteins

Table 3 characterises structure, function, and regulation of the TJ-associated MARVEL proteins (TAMPs) expressed at the BBB, i.e., occludin, tricellulin, and MarvelD3 (expression of the latter reported only once so far [200]). Occludin localises mainly in two-cell contacts [201]; it is found ubiquitously in TJs and is often used as a TJ marker protein [202]. Tricellulin (in particular, tricellulin a), is expressed with markedly lower total expression level (Figure 5, dashed box) [50] but is highly enriched in tricellular contacts [203] (Figure 2C). TAMPs show four transmembrane domains, cytosolic termini, one intra-, and two extracellular loops [22] (the second containing a conserved intramolecular disulfide bridge [133,204]. The MARVEL (MAL and related proteins for vesicle trafficking and membrane link) domain comprises all transmembrane domains and loops [205] (Figure 2D) that form cholesterol-rich microdomains in plasma membrane appositions [206]. The TAMPs are involved in the formation of TJs [207,208] by interacting with claudins, but occludin and tricellulin do not bind each other [55], although they support TJ-strand branching and stabilise epithelial barrier integrity [209]. Tricellulin may also occur at bicellular contacts [210] and can partially compensate for occludin [22], thus contributing to paracellular tightness [211]. Occludin and tricellulin are redox-sensitive and regulate bicellular and tricellular TJs under oxidative or reducing conditions [133,204].

The exact function of occludin is still unclear. There is no evidence for a direct barrier function [212,213], although heterophilic and homophilic oligomerisation do occur [55,204], the latter via its cytosolic C-terminal coiled-coil domain [214]. This domain is involved in macromolecule flux through TJ barriers [215], ZO-1 association [215,216], and their proteins are targets of various protein kinases [217,218]. Multiple phosphorylation [202] is demonstrated via a molecular weight shift in electrophoresis [219]. These and other modifications [220] are mediated by different signalling pathways (relevant for normal and pathological conditions) and strongly point at a regulatory function, e.g., via interaction with claudins and/or ZO proteins (Figure 2D) [205]. The number of studies that exploit these pathways for new medical applications is increasing [141,221]. There is wide agreement that occludin is involved in the regulation of the TJ permeability [212,222]. 

Tricellulin fulfils its general function, sealing the gap at the contact points (Figure 2C) of three membranes. In the BBB, primarily single endothelial cells form distinct capillary segments by themselves; thus, three separated membrane patches from two different cells meet in one point (three-cell-like contacts, Figure 2A, right) [203,223]. The protein oligomerises homophilicly, creates tricellular TJs [133], and tightens this area (in epithelial cells) for molecules < 10 kDa without affecting ion permeability [211]. Comparing bi- and tricellular contacts, it is concluded that the expressed amount of sealing TJ proteins (claudin-5 and -11) in bicellular TJs is about two orders of magnitude higher than that of tricellulin [50]. This suggests that the BBB function is quantitatively determined by two-cell TJs, which prevent permeation of small and large molecular weight compounds [63], rather than by tricellular TJs that withhold larger molecules [211,224].

In three-cell(-like) contacts of brain endothelial cells, tricellulin specifically concentrates and colocalises with angulin-1/LSR (lipolysis-stimulated lipoprotein receptor) [203], a type I transmembrane protein with a single Ig-like domain [225]. Tricellulin and angulins form so-called central sealing elements [226] laterally in three-cell(-like) contacts (Figure 2C). For delivery of antisense oligonucleotides (5.3 kDa) through the BBB, these TJs can be modulated by recombinant angubindin-1, a *Clostridium perfringens* iota-toxin, which binds to angulin-1 [224]. Angulin-1 knockout mice exhibit embryonic lethality [227] with BBB failure [228], which is not known from tricellulin deficiency. These and other observations support the assumption that angulin-1, and not tricellulin, could be essential for sealing the three cell-like contacts in cerebral capillaries. In epithelial cells, angulin-1 forms tricellular contacts, even in tricellulin- or claudin-deficient cells [229]. 

**Table 3:** Tight junction-associated MARVEL-proteins (TAMPs) expressed at the blood-brain barrier.

Expression	Function	Structure/Interactions	Regulation/Signalling
**Occludin**			
- gene *OCLN*, chromosome 5 (human), -13 (mouse) - expression < Cldn5 [50] - increased expression in co-culture of BEC with astro-/pericytes [230], neurons [231] - half-life 6.2 h [189] - KO mouse: TJ-morphology unchanged, calcification of brain [212] - Ocln/Tric-double KO: lowers TJ-strand branches/ barrier integrity [209]	- TJ-regulation postulated [192] - redox sensor in TJs [204] - facilitates TJ branching/barrier tightness [55,209] - C-terminal CC-domain required for maintenance and regulation of macromolecule flux through TJs [215] - regulates centrosomes in cortex genesis [232] - regulates apoptosis via caspase-3 transcription [233] - controls HIV-transcription [234], glucose uptake/ATP-synthesis [235] of BBB pericytes - Ocln/caveolin-1/Alix-complex regulates HIV-permeation through BBB [192] - required for cytokine-mediated signal transduction [236]	- human: 522 aa; M.W. 59.1 kDa (polyphosphorylated ≤ 65 kDa [219]); pI 5.77 - cytosolic N-/C-termini 66/257 aa; ECL1 46 aa (11 Tyr, 19 Gly—potentially hydrophobic interactions, flexibility); ICL 11 aa; ECL2 48 aa (2 Cys, disulfide bridge, hypoxia-/redox-sensitive) [204] - interactions: homophilic *trans*, *cis* (CC do- main dimerises [214]); heterophilic *cis* Cldn1, MD3/*trans* Cldn1, -5 [55], -25 [50] - 3D-structures: cytosolic C-terminal region [237], complex ZO-1 (PDZ3-SH3-U5-GuK)/ Ocln (CC-domain) [222] - CC-domain binds ZO-1 (SH3-hinge-GuK) [216], possibly interacts with ZO-1 [215] - peptide sequence of CC-domain associates PKC ζ, Tyr-kinase c-Yes, PI3K, connexin 26 [218] - MARVEL-domain: in membrane appositions, cholesterol-rich microdomains [206]; mediates interaction of TJs with membrane lipids, *cis*-oligomerisation via Cys and membrane insertion [238]	- Tyr398, Ser408: high-conserved phosphorylation sites for PKCs, CK2, Tyr-kinase Src [217] - thrombin: Tyr-phosphorylation, Ocln-ZO-1-/TJ disruption, BBB leakage; angiopoietin-1 inhibits this Tyr-phosphorylation, stabilises TJs [239] - VEGF-activated atypical PKC opens BBB [240] - VEGF/hypoxia activate PLCγ, PI3K/Akt, PKG: rearrange Ocln, ZO-1, -2; open BBB [241] - EGFR-activation: p38 MAPK/NFκB signal pathway reduce Ocln expression [242] - ubiquitinated by E3A Nedd4-2 [243]/Itch [244] (prevented by γ-secretase blockade [245] - KD of E3A MARCH3 tightens BEC barrier, induces Ocln-/Cldn5 by FoxO1 deactivation [246] - reduction: TGF-β via MMPs [93], IL-17 [247] - degradation: MMP [248,249], calpain (Zn^2+^-dependent) [250], proteasome [244,251] - microwave radiation: reduced Ocln/Ocln-ZO-1 binding, TJ broadening/fracture, BBB opening (VEGF/Flk-1-ERK Tyr-phosphorylation mediated) [252] - ischemia/reperfusion: Ser490 phosphorylation/ ubiquitination via VEGFR2 [253] - Netrin-1 protects BBB, activates Kruppel-like factor 2/Ocln path (ischemia/reperfusion) [221] - hypoxia: MMP9 caused Ocln rearrangement in TJs, BBB leakage [254] - diet-induced diabetes: Ocln/ZO-1 down, BBB leak; lessened by carboanhydrase inhibitor [141] - autistic children: serum Ocln increase [126]
**Tricellulin** (*MARVEL domain-containing protein 2*)			
- gene *MARVELD2*, chromosome 5 (human), 13 (mouse) - particular isoform Tric a [50] - expression <Ocln, <<Cldn5 [50] - brain [223], retina [203,255] - membranal in tricellular [256], bicellular cell contacts [257], likewise nuclear, perinuclear localised [223] - KO mouse: hearing loss, degenerated cochlea hair cells [258] - Tric/Occl-double KO lowers TJ-strand branches/ barrier integrity [209]	- sealing of macromolecules but not ions in tricellular TJs [211] - regulates H_2_O-permeability [259] - role in regulating blood-cerebrospinal fluid barrier [255] - facilitates TJ branching/barrier integrity [55,209] - redox-regulation in TJs [260]	- mouse: 558 aa, M.W. 64.2 kDa; pI 7.21 - cytosolic N-/C-terminal 194/196 aa; very short ECL1/ECL2 8/16 aa; ECL2: disulfide bridge, hypoxia-/redox-sensitive [260] - homophilic: *cis* interaction in 2- and 3-cell TJs; *trans* in 3-cellular TJs [55,260] - heterophilic interaction: *cis* Cldn1, -3, -5, MD3; *trans* Cldn1, -5 [55] - continous P-face strand network in 3-cell TJs [260]; intensifies Cldn1 TJs [55] - C-terminal CC-domain: crystal structure (2.2 Å), dimer with polar interface [261] - angulins bind/recruit Tric in TJs [225] - N-terminus associates dynamin-binding protein (=scaffold protein Tuba) [262], human plasminogen [263]	- ubiquitination by Itch [264] - MAPK-, PKC-activation causes nuclear localisation in weakly differentiated tissue [265] - toxin ESX-1 secretion-associated protein EspG1 reduces expression [266] - induction by mirRNA-203 (microRNA binding motif on Tric) inhibitor, weakening Pb-induced blood-cerebrospinal fluid barrier leak [255] - down-regulated: interleukin-13 (via IL-13-receptor α2) [267], choleratoxin [268] - degradation: by MMP2/3 [269] - apoptosis: degraded at Asp487, Asp 441 (C-terminal CC-domain, caspase cleavage) [270] - OGD: Tric down in BEC [200] - increase in cortex: autism spectrum disorders (Cldn3, -5, -12) [271]
**MarvelD3** (*MARVEL domain-containing protein 3*)			
- gene *MARVELD3*, chromosome 16 (human) - KD retards TJ formation [22]	- may partially replace Ocln, Tric [22]	- human 401 aa, M.W. 44.9 Da; pI 8.84; ECL1/2 24/39, N-/C-terminal 226/39 aa - *cis* binding: MD3, Ocln, Tric, Cldn1, -5 [55]	- down-regulated by OGD in bovine BEC [55]

aa, amino acid; BBB, blood-brain barrier; BEC, brain endothelial cell; CC, cytosolic C-terminal coiled-coil (OCEL, ELL) domain; *cis* interaction, cf. Figure 4B; CK, casein kinase; Cldn, claudin; EGFR, epidermal growth factor receptor; ECL/ICL, extracellular/intracellular loop; E3A, E3 ubiquitin ligase; FoxO1, forkhead box O1; KO/KD, knockout/-down; MAPK, mitogen-activated protein kinase; MARVEL, MAL and related proteins for vesicle trafficking and membrane link; MARVEL-domain, transmembrane domain 1–4, ECL1, ICL, ECL2; MD3, MarvelD3; MMP, matrix metalloproteinase; M.W., molecular weight; NFκB, nuclear factor κB; Ocln, occludin; OGD, oxygen/glucose deprivation; P-face, protoplasmic face; pI, isoelectric point; PI3K, phosphoinositol 3-kinase; PK, proteinkinase (PKB/Act, PKC, PKG); PL, phospholipase; TGF, transforming growth factor; TJ, tight junction; *trans* interaction, cf. Figure 4B; Tric, tricellulin; VEGF(R), vascular endothelial growth factor (receptor); ZO-1, *zonula occludens* protein 1.

### 3.3. Junctional Adhesion Molecules

The JAMs belong to the immunoglobulin superfamily [272], form one transmembrane domain, and are connected to the cytoskeleton via the binding of their short C-terminus to the PDZ3 domain of ZO-1 [273]. The N-terminal extracellular domain contains two immunoglobulin-like loops which can interact homo- and heterophilically in *cis* (with proteins of the same endothelial cell) or *trans* (e.g., with proteins on blood cell proteins) [272,274,275] (Figure 4B). Mainly JAM-A (JAM-1) [276], JAM-C (JAM-3) [277], and the endothelial-cell-selective adhesion molecule (ESAM) are found in TJs of the BBB [278,279] and support barrier features. JAM-A binds to integrin α-V β3 in *cis* [280] and integrin α-L of leukocytes in *trans* [281], and JAM-C binds to integrin α-M (*trans*, leukocyte) [282]). JAMs support the correct localisation of other junctional proteins (e.g., claudins) at the TJs [283] and stabilise cell barriers [284]. They are also involved in the regulation of cell contact formation, cellular migration, and mitosis, and, in this way, take part in barrier formation, angiogenesis and cerebral homoeostasis [285]. JAM-A regulates barrier properties by promoting the expression of C/EPB-α, a transcription factor regulating claudin-5 [125]. ESAM seems to be involved in the endothelial tube formation [286] (Figure 2A), as well as in the extravasation of white blood cells [287]; however, deactivation of the ESAM gene does not change the vascular permeability in mouse brain [288].

### 3.4. Cytosolic Tight Junction-Associated Proteins

The guanylate kinase homologous (MAGuKs [289]) ZO-1 (TJP1, 225 kDa [290]), as well as N-terminally truncated ZO-2 (TJP1, 160 kDa [291]) and ZO-3 (TJP3, 130 kDa [292]), are the most important membrane-associated proteins on the cytosolic TJ sides in the BBB. ZO proteins are involved in the formation and function of adherens- [293] and gap junctions [294]; consequently, ZO-1 is often used as cell contact marker [50]. In addition, ZO proteins are included in the regulation of cytoskeletal organisation, the establishment of cell polarity, and signalling to and from the nucleus [24]. They constitute the scaffold of TJs via multiple binding areas (in ZO-1: _NH2_PDZ1–PDZ2–PDZ3–SH3–hinge region–GuK–acidic region–U6 region–proline rich region–ZU5 region_COOH_, Figure 2D). These sections recruit transmembrane TJ-proteins and associate signalling- and structural proteins which, in turn, are involved in the formation, regulation, and/or stabilisation of TJs [295]. PDZ1 associates with claudins, and PDZ2 mediates the dimerisation of ZO proteins and may provide a structural basis for the association of claudins, namely, the formation of TJs [191,296]. PDZ3 and SH3-hinge-GuK bind JAMs and occludin, respectively [216]. SH3 interacts with ZONAB [297]. The hinge region (U5) attracts G-proteins [298], enabling a wide diversity of G-protein-coupled receptors to regulate the BBB [299]. Part of the proline-rich region (amino acids 1151–1371 of ZO-1) allows for the anchoring at the actin cytoskeleton [300]. Nuclear localisation of ZO proteins plays a further role in the signalling of TJ proteins [301,302] (Figure 2D). ZO-1 and ZO-2 can compensate for each other; only double knockdown leads to changes in the localisation of claudins and occludin, resulting in paracellular leakage [293,303,304]. 

Another scaffolding protein of the apical junctional complex is afadin (AF-6, gene AFDN, a multidomain protein with one PDZ, binds JAM-A). It interacts N-terminally with membranal adhesion- and signalling proteins while its C-terminus binds to the actin filament and to actin-binding proteins. Afadin can modulate signalling processes that influence cellular migration, invasion, and apoptosis [305]. Afadin is expressed at the cerebral endothelium [306] and contributes (in cooperation with PI3K/Akt signalling) to neovascularisation [307]. The cellular polarity at the TJs is maintained by the apical polarity complex, which includes the PAR complex (PAR3, PAR6, aPKC [308]) and the basolateral scribble complex (scribble, DLG, LGL). These complexes form networks via several signalling pathways (e.g., small GTPases as RhoA, RAC, and CDC42 [309]; Wnt/β-catenin [310]); their disbalance leads to perturbations in the barrier function [311]. aPKC and RhoA [283], as well as the PDZ-free scaffolding cingulin-like protein 1 (gene JACOP) [312], are involved in the regulation of the endothelial TJ conglomerate.

Summarising the data of the differentiated BBB, claudin-5 is proven to be the most prominent TJ component that bicellularly seals the barrier, probably assisted by claudin-11. The proper morphology and function of the TJ strand network is facilitated by occludin and tricellulin under support of ZO-1. Quantitatively, the sealing capacity is mainly accomplished by two cell (-like) TJs. The barrier function is subject of versatile regulation by signalling pathways relevant under normal and pathological conditions that particularly target claudin-5 and, to an even greater extent, occludin.

## 4. Tight Junctions and Pathologies of the Brain

In Table 4, disease states and diseases are compiled, which are accompanied by BBB impairment and TJ protein involvement. It is a widely held consideration that BBB damage is a consequence of many severe pathological processes of the brain. However, evidence is given that barrier disturbances can also be triggered by peripheral diseases [313] or by mutations of TJ proteins [115,314]. Leakage of the barrier is observed in the early stages of some brain diseases and might even contribute to disease progression [315,316]. Various cerebral diseases, such as ischemia and related disorders, tumours or, inflammatory processes, can lead to BBB disturbances, imbalances of ion-/molecular fluxes, increased extravasation of blood cells, and impaired TJ morphology [317]. Novel BBB models of human-induced pluripotent stem cells have been introduced to study diverse neurological diseases [318]. Details are given in Table 4.

Similar alterations of the barrier are described for neurodegenerative [359] and psychiatric disorders (e.g., schizophrenia, autism spectrum disorder, affective disorders) [330]. In the context of Alzheimer’s disease, angiogenic processes can be triggered by β-amyloid, which result in a loss of microvascular TJ proteins (claudin-1, -5) and increased permeability of the barrier in rodent brain [319,320]. In human stem cell models, claudin-3 and -5 are found up-regulated [321]. In patients with Alzheimer’s disease, plasma claudin-5 levels are increased; the protein has been suggested as a potential biomarker for the diagnosis of AD [360]. 

Multiple sclerosis is characterised by decreased levels of claudin-11 at the BBB of patients [130] and of claudin-3 in a mouse model which exhibits lowered barrier tightness [78]. Another mouse study revealed down-regulation of angulin-1, suggesting that tricellular TJs could be disturbed during multiple sclerosis [228]. The inflammatory cytokine IFNγ safeguards tight junctions of the BBB in a mouse model of multiple sclerosis by up-regulating claudin-5, which could be inhibited by TNFα and IL17 [361], revealing a physiological protective mechanism of the BBB during inflammation.

In a model of amyotrophic lateral sclerosis, reduction of claudin-5, occludin, and ZO-1 in endothelial cells results in microhaemorrhages before motor neuron degeneration and neurovascular inflammatory markers occur, indicating a central contribution to disease initiation [324]. For schizophrenia, a variant in the claudin-5 gene is reported, leading to suppression of claudin-5 [314]. Interestingly, loss of TJ proteins from a damaged BBB can be detected in the peripheral blood of autistic children (claudin-5, -11, occludin) [126], supporting earlier findings that claudin-5 is released from the brain endothelium during disease. Claudin-5 can be transferred to circulating leukocytes, which could support leukocyte transendothelial migration into the CNS [116].

The BBB in brain tumours is widely intact in early stages but breaks down during progression of the tumour [331] with a concomitant diminution of claudin-1, -3, -5, and occludin [78,80,93]. Many different types of brain tumours have been documented, showing large heterogeneity. They arise from primary tumours, such as glioblastoma or astrocytoma, and metastatic tumours frequently originate from breast or lung cancer [362].

Breakdown of the BBB is well documented for ischemic states [9,338], which lead to disorders such as stroke or oedema [363]. At the molecular level, multiple factors are discussed: oxidative/nitrosative stress, metabolic/ionic dysregulation, and/or inflammatory/neurodegenerative processes [364]. Experimental ischemia/reperfusion results in the loss of claudin-1, -3, -12, and occludin, whereas claudin-5 has been found to be up-regulated 3 h after occlusion [50,53]. After 120 h, similar effects have been observed for occludin and ZO-1, but this was also true for claudin-5 in the study in question, suggesting a biphasic time-course [338]. Claudin-5 has been also found in the serum of stroke patients [127]. Studies on protective approaches often report claudin-5 preservation, e.g., by a novel antioxidant, attenuating BBB breakdown via erythroid 2-like 2 nuclear translocation/haem oxygenase 1 signalling stimulation [129], by small molecules [365], or by mesenchymal stem cells [366]. Endothelial reduction of claudin-25 and the subsequent permeability increase for small molecules have been shown for cerebellar haemorrhage [76]. Disturbances of the cerebral circulation are fostered by obesity [345], diabetes mellitus [340], or microangiopathies [342]. BBB leakage is also accompanied by the down-regulation of claudin-12, occludin, and ZO-1 in obesity with concomitant type II diabetes [141], or of claudin-5 and occludin in diabetes [341]. Interestingly, Tie2^+^ macrophages promote endogenous revascularisation in mouse brains after ischemic injury [367], which possibly leads to reconstruction of the BBB.

Infarct progression and oedema formation can be increased by a tight barrier [53]. Absence of claudin-3 and a reduced level of occludin can limit the infarcted and oedematous area. Consequently, TJ modulation has been postulated as an approach to treating stroke and related disorders at least early after onset. There is good evidence that mild trauma resulting in BBB disruption in rats opens a paracellular pathway of approximately 22 nm, consistent with disrupted TJs, leaving adherens junctions intact [368]. In this context, EphA4/Tie2/Akap12 signalling has been reported to limit the expression of claudin-5 and to mediate microvascular dysfunction [128]. On the other hand, trauma-associated brain oedema can be diminished via suppression of claudin-5 after administration of the corresponding siRNA in rats [335] or by a novel inhibitor of claudin-5 interactions [369], which also alleviates ZO-1 degradation [336]. In addition to its direct effect, claudin-5 siRNA can be applied to improve the pharmacokinetics of agents targeting brain diseases [185]. There are other modulators discussed in this context: claudin-5 shRNA [370], monoclonal anti-claudin antibodies [371], and peptides (disclosed by phage display [372,373,374] or derived from sequences of the extracellular loop 1 of claudins [190,199]), which exerted transient BBB opening. The last-mentioned approaches can be generalised to the extent that small agents can be developed according to this principle to open the BBB transiently and size-selectively and provide conditions allowing for the delivery of a wide range of compounds for the treatment of neurodegenerative, neuropsychiatric, and malignant diseases. For the delivery of larger molecules, such as antisense oligonucleotides, the angulin-1 binder angubindin-1 has been applied in vivo. The data demonstrate that not only bicellular but also tricellular TJs may be targeted to improve drug permeation through the BBB [375].

Cerebral inflammation is often induced by infections. Zika or meningitis viruses cause disruption of the mouse BBB and down-regulation of claudin-5 [350,354]. The malaria pathogen *Plasmodium falciparum* elicits vascular permeability, fatal brain oedema, and down-regulation of claudin-5 and occludin [353]. Chronic inflammatory pain induced by peripheral injection of complete Freund’s adjuvant suppressed occludin but up-regulated claudin-3 and -5 with simultaneous opening of the barrier [182]. Various mediators and pathways are involved in alterations of TJ proteins during inflammation. Vascular endothelial growth factor contributes to BBB opening [83,346] by down-regulating claudin-5 [332]. Increased permeability is also caused by pro-inflammatory stimuli such as thrombin [239] or microbial toxins down-regulating occludin and claudin-5 [347]. Barrier strengthening can be achieved via the administration of anti-inflammatory agents, e.g., of angiopoietin-1, which promotes occludin-ZO-1 interaction and stabilises TJs by inhibiting thrombin-induced Tyr-phosphorylation of occludin [239]. 

Reviewing the latest experimental data and the increasing number of clinical studies reveals more and more neurological disorders characterised by BBB involvement. In the vast majority of cases, disturbances of the TJs at the molecular level of claudin-5 are involved, with evidence of pathogenic significance. Modulation of three cellular TJ-proteins provides a novel approach for drug delivery. Human-induced stem cell models offer diagnostic potential in analysing various neurological diseases individually. These advances have led to new diagnostic approaches and will further encourage pharmacological studies.

## 5. Conclusions and Perspectives

Tight junctions are of crucial importance for the BBB, which both directly and indirectly controls the overwhelming majority of the exchange mechanisms between the brain and blood. On the other hand, a functional barrier may also cause problems such as reduced edema drainage in stroke or trauma or insufficient drug delivery to tumours. Further progress in understanding the BBB requires reliable BBB models; previous model investigations often applied non-human cells with dedifferentiated TJ proteins. Recent developments favour models using human primary and induced pluripotent stem cells. For in vivo studies, the prospects of genetically modified mice should be further exploited in the context of TJs. These approaches bear great potential for BBB-related research with high clinical relevance.

Elucidation of the molecular composition of TJs revealed that claudin-5 plays a central role in tightening the BBB, but the TJ does not solely depend on this claudin; it must be considered in concert with other TJ proteins. In particular, the impact of claudin-11 and tricellulin/angulin requires further experimental and clinical studies. Numerous signalling pathways relevant to the BBB have been clarified, and above all, we have described the role of these in pathologies, as is increasingly being shown clinically. Claudin-5 can be regulated directly, but more often, indirect regulation via occludin is observed. More insight into these processes will open up new diagnostic and therapeutic perspectives.

Nevertheless, the exact role that the BBB plays is still unclear in many CNS diseases, especially the extent to which it itself can cause cerebral dysfunction. Generally, its opening is considered to be a consequence of disease progression, but there is evidence that the BBB disruption is pathogenetic early in the development of disorders. We confidently expect that further molecular mechanisms and protein structures will be disclosed to advance our understanding of TJ biology and to be translated into new therapeutic approaches.

## Figures and Tables

**Table 4:** Pathologies, alterations of permeability and of tight junction proteins at the blood-brain barrier.

Type of Disorder	Leakage of Blood-Brain Barrier	Tight Junction Alteration
neurodegeneration	- m. Alzheimer mouse model [319,320]; iBEC layer, mutant transfected [321]	- ß-amyloid triggered angiogenesis → Cldn1, -5 down-regulated [319,320] → Cldn3, -5 up-regulated [321]
	- multiple sclerosis [322] - EAE [228]	→ down-regulated: Cldn3 (EAE) [78], Cldn11 (patient, EAE/mouse) [130] → angulin-1 down, 3-cell contacts [228]
	- amyotrophic lateral sclerosis [323]	- mouse model → BCSFB: loss of Cldn5, Ocln, ZO-1 [324]
	- m. Parkinson, extravasation in striatum [325]	- rat model → Cldn5, Ocln, ZO-1 up-regulated (*substantia nigra*) [326]
	- chorea Huntington, human and mouse model [327,328]	→ Occl, ZO-1 reduced in iBEC [328]
epilepsy	- cainic acid-induced seizures, temporal lope epilepsy [329]	- resected brain → Cldn5, Ocln, ZO-1 reduced [329]
psychiatric disorders	- schizophrenia, autism spectrum disorder (ADS), affective disorders [330] - ADS, cortex	→ Cldn12, Ocln, ZO-1 down-regulated [330] → Cldn3, -5, -12, Tric up [271]
	- schizophrenia associated with Cldn5 gene variant [314]	→ lessens Cldn5 in BEC; antipsychotic drug enhances Cldn5 [314]
brain tumours	- advanced tumour grades [78,93,331]	→ reduction of Cldn1, -3, -5, Ocln (glioblastoma) [78,80,332]
traumatic brain injury	- rat model [333] - patients [334] - Cldn5-si/shRNA enhanced leakage, reduced swelling [335]	→Ocln, ZO-1 reduced [333] → Cldn5 reversibly down-regulated [335] → ZO-1 degradation [336]
ischemia/stroke	- acute ischemic stroke, human [337] - ischemia/reperfusion, mouse [129]	- at clinical worsening → Cldn5, Cldn5:ZO-1 ratio increased in blood [127]
	- middle cerebral artery occlusion [228,338]	→ 3 h: Cldn1, -3, -12, Ocln dropped, but Cldn5 rose [50,53]; 5 d: Cldn5, Ocln, ZO-1 reduced [338] → angulin-1 down, 3-cell contact [228]
	- hypoxia/glucose lack, BEC [200]	→ Cldn5, Ocln, ZO-1 down [339]
	- haemorrhage [76,104]	→ Cldn25 down-regulated (BEC) [76] → Cldn3, -5 down; improvement/reduced leak by anti-malaria drug [104]
*- reinforced by*	- d. mellitus [340] - BRB leakage in murine diabetic retinopathy [341] - microangiopathy: small vessel disease, stroke imaging [342]; iBEC layer, mutant transfected [343]	→ Cldn5, Ocln depressed [341] → Cldn5 and ZO-1 expression reduced (autopsy samples) [344] → Cldn5-, Ocln-junctions affected [343]
high-fat diet	- diet-induced obese diabetic mice [141]; obesity [345]	→ Cldn12, Ocln, ZO-1 reduced [141]
inflammation	- thrombin-caused, BEC layer [239] - astrocyte-derived VEGF [346]	→ Ocln-ZO-1 binding lost [239] → Cldn5 decrease in BEC [332]
	- peripheral (CFA) [182,347,348] - pancreatitis [349]	→ Ocln down/Cldn3, -5 up (CFA) [182] → degradation of ZO-1, Cldn5 [349]
infection	- Zika virus in mice [350]	→ Cldn5 down [350], via miR-101-3p [351]
	- bacterial pertussis toxin [347]	→ ZO-1 down (BCSFB) [352]
	- *Plasmodium falciparum* [353] - *Neisseria meningitidis*, iBEC [354] - long COVID patients [355]	→ Cldn5, Ocln down (mouse) [353] → Cldn5, Ocln down (cell layer) [354] → Cldn5 down (mouse) [356]
hypertonia	- acute in rat [357]	→ Cldn3, -5, -12 depression [357]
alcohol abuse	- hippocampal IgG extravasation [358]	→ Cldn5 down [358]
microcephaly	- small molecule flux higher [115]	→ human Cldn5-G60R (ECL1) [115]

BCSFB, blood-cerebrospinal fluid barrier; BEC, brain endothelial cells; BRB, blood-retina barrier; CFA, complete Freund’s adjuvant; Cldn, claudin; EAE, experimental autoimmune encephalitis; ECL, extracellular loop; iBEC, BEC induced from human embryonic stem cells; Ocln, occludin; VEGF, vascular endothelial growth factor; ZO-1, *zonula occludens* protein 1.

## Supplementary Materials

The following supporting information can be downloaded at https://www.mdpi.com/article/10.3390/ijms25115601/s1.
