# Supplementary figures and images for: The Basic Requirement of Tight Junction Proteins in Blood-Brain Barrier Function and Their Role in Pathologies
Source: Int J Mol Sci. 2024 May 21;25(11):5601. doi: 10.3390/ijms25115601 (PMC11172262; doi:10.3390/ijms25115601)

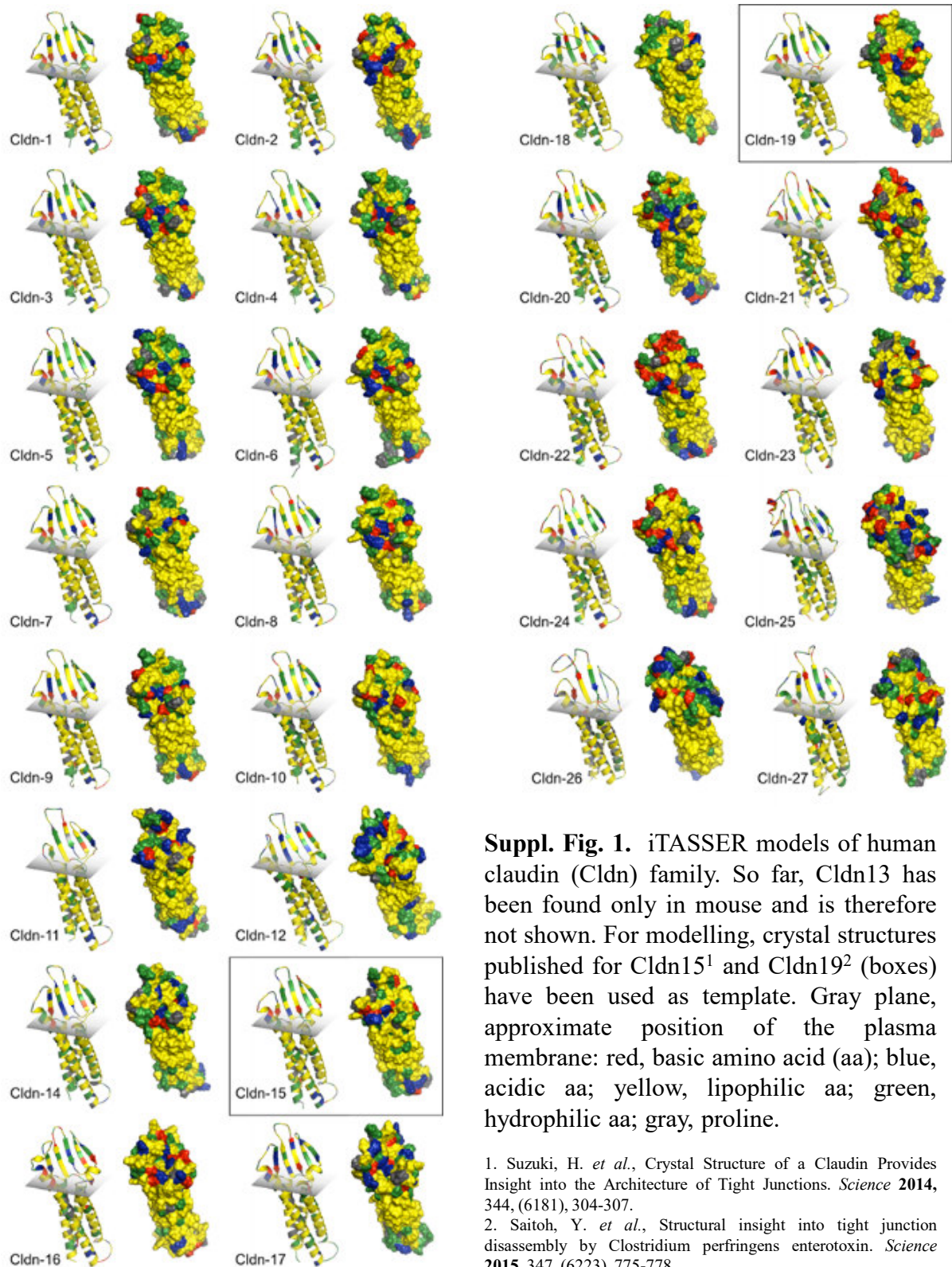

*Acknowledgement: The authors wish to thank Dr. N. Gehne for providing this figure.*

Supplement: Supplementary file 1 [file ijms-25-05601-s001.zip › ijms-2887859-supplementary.pdf]
